# Transmission of respiratory infectious diseases based on real close contact behavior in an emergency room

**DOI:** 10.1016/j.idm.2025.07.004

**Published:** 2025-07-05

**Authors:** Bing Cao, Haochen Zhang, Nan Zhang

**Affiliations:** Beijing Key Laboratory of Green Built Environment and Energy Efficient Technology, Beijing University of Technology, Pingleyuan100, Chaoyang District, Beijing, 100124, China

**Keywords:** Healthcare worker (HCW), Nosocomial infection, Emergency room, Close contact, Respiratory infectious disease, COVID-19

## Abstract

**Background:**

The risk of transmission of respiratory infectious diseases in emergency rooms is high, posing a severe threat to the health of healthcare workers (HCWs).

**Methods:**

The study was conducted in an emergency room of a medical school at a university in Hong Kong during a clinical skills competition. A total of 19,246 s of video surveillance data were collected, recording the treatment of three types of patients (P1: infusion patient, P2: critically ill patient, P3: agitated patient). Taking coronavirus disease 2019 (COVID-19) as an example, a multi-route transmission model was established to assess the infection risk for HCWs and the effectiveness of various interventions.

**Results:**

The average distances between HCWs and patients during the treatment of P1, P2, and P3 were 0.8 (25–75 percentile: 0.6, 1.1) m, 1.0 (0.8, 1.2) m, and 0.5 (0.4, 0.7) m, respectively. When treating P2, due to intubation procedures, the hourly risk of infection was highest at 43.4 % if no HCWs wore masks, which was 5.1 and 3.1 times higher than it during treatment of P1 (8.5 %) and P3 (13.9 %), respectively. During the treatment, without mask protection, the average hourly infection risk for nurses was 11.0 % (P1), 41.2 % (P2), and 16.8 % (P3), which was 1.8 times (P1), 0.9 times (P2), and 1.5 times (P3) that of doctors. If HCWs wear N95 respirators and surgical masks throughout, the total infection risk can be reduced by 94.7 % and 53.9 %, respectively. Increasing the ventilation rate from 1 ACH to 6 ACH reduced the infection risk through long-range airborne transmission by 43.8 % (P1), 36.1 % (P2), and 31.6 % (P3), with a total infection risk reduction of 2.4 % (P1), 5.6 % (P2), and 1.6 % (P3), respectively.

**Conclusions:**

The findings of the study provide a scientific support for the precise prevention and control of respiratory infectious diseases under different treatments in emergency rooms.

## Graphical abstract

**Figure:**
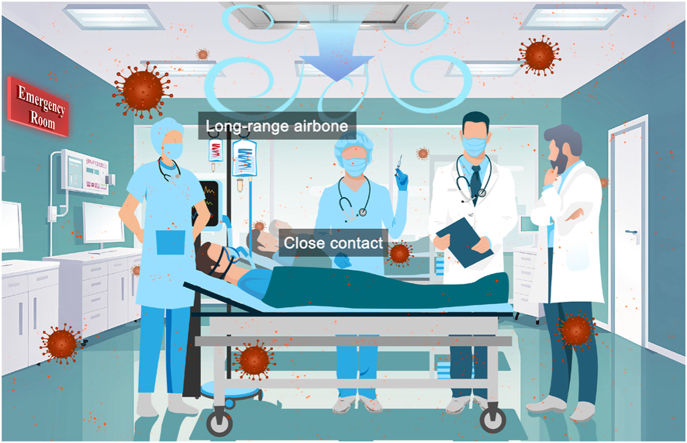

## 1 Background

Respiratory infectious diseases are prevalent worldwide, with healthcare facilities being high-risk environments (Richterman et al., 2020). The coronavirus disease 2019 (COVID-19) pandemic presented enormous challenges to healthcare systems.

By the end of 2022, China had more than 1,000,000 healthcare institutions and 9,659,000 healthcare workers (HCWs) (2022 China Health Statistical Report. 2023). In some countries, HCWs accounted for 3.5 %–20 % of COVID-19 infections (Ali et al., 2020). In China, the United States, and Italy, HCWs accounted for an average of one-tenth of those infected (Sahu et al., 2020). Although HCWs made up less than 0.7 % of China's population, their infection rate was ten times higher than that of others (Ha, 2020). The World Health Organization (WHO) estimated that from January 2020 to May 2021, approximately 115,000 HCWs worldwide died from COVID-19, with a mortality rate twice that of the general population (WHO, 2021). However, the underreporting of cases may lead to an inability to determine the 'true' incidence rate of the disease (Kalachev et al., 2024).

Nosocomial infection is defined as an infection acquired in hospital by a patient who was admitted for a reason other than that infection, and in whom the pathogen was not incubating at the time of admission (Carter et al., 2020). The prevalence of nosocomial infection during SARS (severe acute respiratory syndrome) in 2003 and MERS (Middle East respiratory syndrome) in 2012 was 36 % and 56 %, respectively (Zhou et al., 2020). In the first three months of COVID-19 in China, 44 % of confirmed patients contracted the virus in hospitals (Wang et al., 2020; Zhou et al., 2020). Two studies in the United States counted 83,775 (Billock et al., 2022) and 5500 (Hartmann et al., 2021)HCWs diagnosed with COVID-19, of which 52.0 % and 44.0 %, respectively, were caused by health care exposure. A study in Milan, Italy (Mandić-Rajčević et al., 2020) showed that 60 % of the 172 HCWs diagnosed at the beginning of the epidemic were infected due to medical exposure. Therefore, HCWs in hospitals are at extremely high risk of contracting respiratory infectious diseases. At the same time, HCWs have a higher risk of developing severe illness from lower respiratory tract infections compared to upper respiratory tract infections (Nakagawara et al., 2022).

The emergency room is one of the highest-risk areas within hospitals (Piapan et al., 2022). Frequent contacts between HCWs and patients with unidentified infectious diseases in emergency rooms expose HCWs to a high risk of respiratory disease transmission. Many studies have reported outbreaks in emergency rooms caused by SARS-CoV (Chen et al., 2004), MERS-CoV (Cho et al., 2016), and SARS-CoV-2 (Cooper et al., 2023; Leal et al., 2023), which have led to community spread. For example, 19 HCWs were infected by SARS after close contact with patients in an emergency room at National Taiwan University Hospital in 2004 (Chen et al., 2004). In 2015, an outbreak of MERS in an emergency room at Seoul's Samsung Medical Center led to eight HCWs infections (Cho et al., 2016). In the early stages of the COVID-19 pandemic, 40 HCWs were infected in a hospital in Wuhan, with seven cases from the emergency department (Billock et al., 2022). Therefore, infection prevention and control in emergency rooms is crucial.

SARS-CoV-2 is highly transmissible (Liu, Gayle, et al., 2020), primarily spreading through close contact (short-range inhalation and facial mucous deposition) and through long-range airborne transmission (Greenhalgh et al., 2021; Spina et al., 2020). Close contact behaviors, such as interpersonal distance and relative facial orientation, directly influence the risk of respiratory disease transmission (Gokmen et al., 2021; Welsch et al., 2021). The close contact between HCWs and patients, along with high personnel mobility in emergency rooms, significantly increases transmission risk. Poor ventilation can lead to widespread outbreaks through long-range airborne transmission (Azimi et al., 2021; Duval et al., 2022; Wang et al., 2021). Understanding the behaviors of HCWs and patients in emergency rooms is critical for effective disease prevention and control. However, few studies have examined the impact of real close contact behaviors on infection risk in emergency room settings.

This study analyzed video data from a clinical skills competition in an emergency room of a medical school in Hong Kong. Close contact behaviors such as close contact rate, duration per close contact, talk rate, interpersonal distance, relative facial orientation and mask-wearing rate were collected. A multi-route respiratory disease transmission model was developed. Taking COVID-19 as an example, the infection risks to HCWs during the treatment of different patient types (infusion patients, critically ill patients, agitated patients) and the effectiveness of mask-wearing and indoor ventilation were evaluated.

## 2 Methods

### 2.1 Transmission route of SARS-CoV-2

**Fig. 1:**
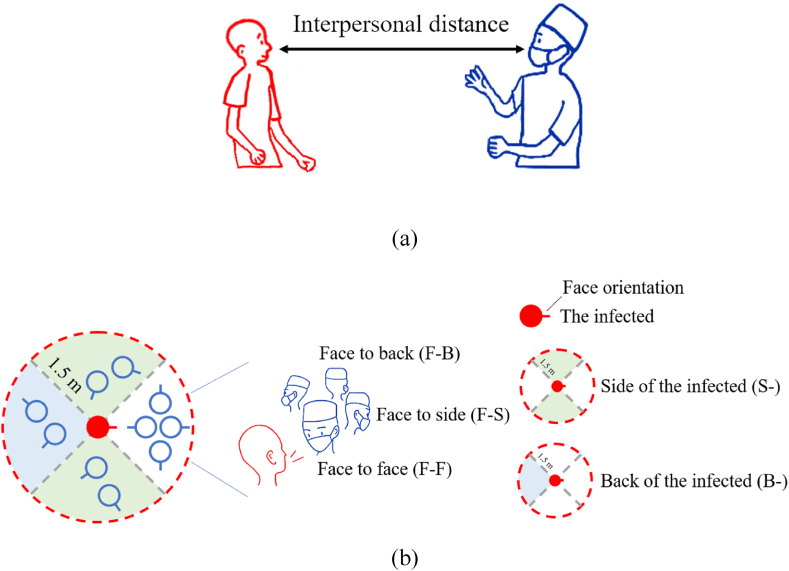
Close contact behavior. (a) Interpersonal distance; (b) Relative facial orientation.

Close contact and long-range airborne transmission are the main routes of SARS-CoV-2 transmission. Fine aerosols exhaled by an infected individual can be inhaled by a susceptible individual through short-range airborne transmission, while large droplets can directly deposit on the facial mucous membranes of the susceptible individual, a process referred to as close contact transmission. The World Health Organization (WHO) and the Centers for Disease Control and Prevention (CDC) define close contact as contact where the interpersonal distance is less than 1.5 m (WHO, 2022; CDC, 2022; Liu et al., 2017), encompassing both communicative and non-communicative behaviors, which contribute to a heightened risk of infection. Close contact behaviors (Table 1), including close contact rate, duration per close contact, talk rate, interpersonal distance (Fig. 1a), relative facial orientation (Fig. 1b) and mask-wearing rate, directly influence the risk of close contact transmission.

**Table 1:** Definition of close contact behavior.

Close contact behavior	Definition
Close contact rate	Ratio of the close contact time to the total recording time
Duration per close contact	The duration of each close contact between HCWs and patient.
Talk rate	Ratio of the talking time to the total recording time
Interpersonal distance	Distance between the noses of HCW and patient
Relative facial orientation	Relative facial orientation between the faces of HCW and patient (Fig. 1b), including face-to-face (F-F), face-to-side (F-S), face-to-back (F-B), side of the infected (S-), and back of the infected (B-)
Mask wearing rate	Ratio of the mask wearing time to the total recording time

**Fig. 2:**
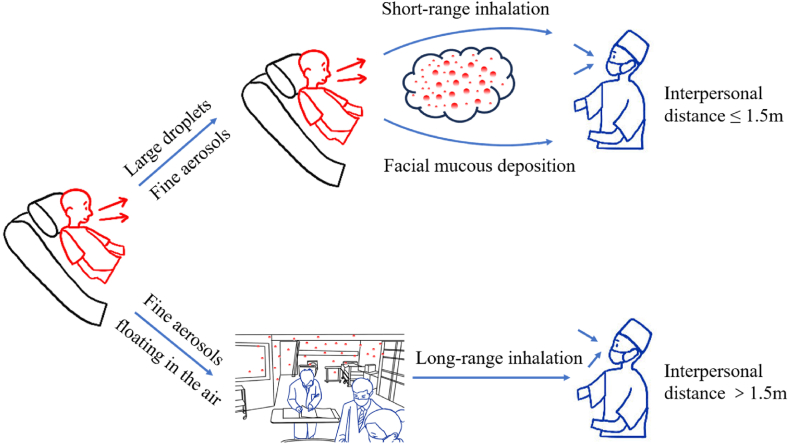
Transmission routes of SARS-CoV-2 in the emergency room.

Fine aerosols (<5 μm) can remain airborne for extended periods and disperse over greater distances, contributing to long-range airborne transmission in hospital wards (WHO, 2023; Groma et al., 2023; Stern et al., 2021). Fomite transmission has been deemed to play a limited role in the spread of SARS-CoV-2 (Goldman, 2020), therefore, has not been considered in our study. Fig. 2 illustrates the primary transmission routes of SARS-CoV-2 within the emergency room setting.

### 2.2 Data collection

**Fig. 3:**
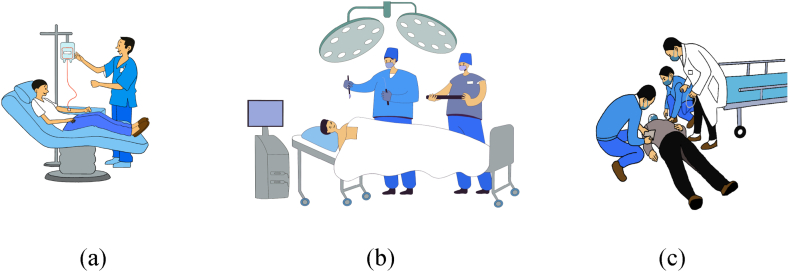
Three treated patients. (a) P1: infusion patient (mannequin); (b) P2: critically ill patient (mannequin); (c) P3: agitated patient (real person).

We obtained video data from a clinical skills competition at a medical school in Hong Kong, encompassing 19,246 s of data depicting interactions between HCWs and patients in an emergency room setting. This study specifically focused on close contact among patients, doctors, and nurses, documenting their behaviors in such scenarios. The video data were categorized into six groups, each consisting of 3 doctors (one attending doctor and two treating doctors), 2 nurses, and 3 patients. For statistical convenience, the 13 doctors were abbreviated as D1-D13, and the 12 nurses as N1-N12 (Table 2). Among 25 HCWs, 16 were male and 9 were female. The same attending doctor (D13), responsible for overseeing the treating doctors and nurses, as well as being accountable for the patients' treatment plans and outcomes, appeared in all six episodes of videos. Five HCWs in each episode treated three patients in different conditions by sequence. The first patient (P1) was a mannequin, lying in a hospital bed, routinely infused (infusion, blood pressure measurement, heart rate monitoring). Doctors were mainly responsible for observing and recording the patient's condition and occasionally assisting the nurses, who primarily performed the infusion and other operations on the patient. The second patient (P2) was also a mannequin, lying in a hospital bed with critically ill (chest compressions, endotracheal intubation). Due to the urgency of the situation, all doctors and nurses treated the patient together, including using equipment and cooperating in completing chest compressions. The third patient (P3) was a real person, who was agitated (fall, emotional agitation, electrocardiographic monitoring). When the patient fell, the doctors and nurses immediately worked together to help and take care the patient back to the bed. The doctor then continued to record the patient's condition while the nurse fixed the patient's body with straps, calmed him, and continued to monitor his vital signs (Fig. 3).

**Table 2:** Basic information for 6 episodes of video.

Group	Duration (s)	Patient	Doctor			Nurse	
1	4575	P1 to P3	D1	D2	D13	N1	N2
2	2234	P1 to P3	D3	D4	D13	N3	N4
3	4317	P1 to P3	D5	D6	D13	N5	N6
4	2997	P1 to P3	D7	D8	D13	N7	N8
5	2584	P1 to P3	D9	D10	D13	N9	N10
6	2539	P1 to P3	D11	D12	D13	N11	N12

**Fig. 4:**
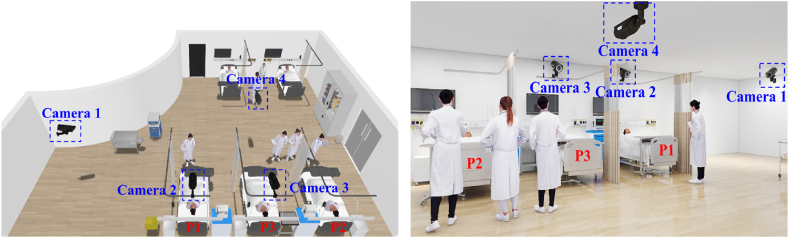
Layout of the emergency room and positions of cameras.

The emergency room in this study measured 13.0 × 10.0 × 2.8 m. The room was equipped with four fixed cameras (Fig. 4), ensuring that each person could be captured by at least two cameras.

The video data were analyzed second by second by six trained students. Interpersonal distance was estimated based on the dimensions of the hospital beds and other items in the emergency room (Zhang et al., 2019). The video analysts recorded each close contact behavior of HCW and patient in a standardized format (Table 3).

**Table 3:** Form for data collection on close contact behaviors.

Target individual^a^	Time^b^	Close contact individual^c^	Relative facial orientation	Interpersonal distance (m)	Mask^d^
N7	4:23:15	P3	F-F	1.2	Y
N7	4:23:16	P3	F-F	1.2	Y
N7	4:23:17	P3	F-F	1.2	Y

a ID of the individuals being tracked (Table 2).

b Cumulative time of the video.

c ID of the person who closely contacted with the target person.

d Whether the target individual wore masks: Y for wearing, N for not.

The final data were cross-checked by a video analyst (first author) every 6 min, and a total of 2200 data points were reviewed, yielding an accuracy rate of 95.1 %.

### 2.3 Exposure calculation

#### 2.3.1 Close contact transmission

Assuming that the three patients in the emergency room were the infected. Two respiratory activities including breathing and talking were considered in the study. Previous research obtained virus generation velocity being 4.5 × 10^−2^ viral RNA loads/s for fine aerosols (<5 μm) during breathing, 1.0 viral RNA loads/s for fine aerosols (<5 μm) during talking, and 7.4 × 10^−2^ viral RNA loads/s for large droplets (≥5 μm) during talking using a G-II exhaled breath collector (Coleman et al., 2021).

In short-range airborne and large droplets transmission, the viral exposure rate decreases with increasing interpersonal distance, defined as distance attenuation. Additionally, the decrease in exposure related to relative facial orientation is termed face orientation attenuation. The close contact transmission attenuation coefficient $\eta_{E}\left(d,o\right)$ is defined as the product of the distance attenuation coefficient ($\eta_{E}\left(d\right)$) and the relative facial orientation attenuation coefficient ($\eta_{E}\left(o\right)$), calculated as follows:

(1) $$\eta_{E}\left(d,o\right)=\eta_{E}\left(d\right)\cdot \eta_{E}\left(o\right)$$

where E represents transmission route, including short-range inhalation (*I*) and facial mucous deposition (*D*); d denotes interpersonal distance; o represents relative facial orientation. Distance attenuation coefficient, $\eta_{E}\left(d\right)$, is defined as the ratio of the exposure rate of a susceptible individual at different interpersonal distances to the viral generation rate of the infected individual during the face-to-face interaction. Similarly, the facial orientation attenuation coefficient, $\eta_{E}\left(o\right)$, is defined as the ratio of the exposure rate for a relative facial orientation to the exposure rate during face-to-face contact at the same distance (Liu et al., 2022). Both the distance and facial orientation attenuation coefficient were derived from previous CFD simulations (Wei et al., 2023).

Considering the differences in aerosol generation rates during talking and breathing, the viral exposure rate $V_{E}$ (viral RNA loads/s) for a susceptible individual during close contact with an infected individual can be calculated using the following equation.

(2) $$V_{E}\left(j\right)=\sum_{s}\left(g_{b,s}\left(i\right)\left(1-\theta \right)+g_{t,s}\left(i\right)\cdot \theta \right)\cdot \eta_{E}\left(d,o\right)$$

where s represents aerosol size, categorized into fine aerosols (<5 μm) and large droplets (≥5 μm); *i* and *j* denote the infected individual and the susceptible individual, respectively; $g_{b}$ and $g_{t}$ represent the viral generation rates during breathing and talking, respectively (viral RNA loads/s); θ denotes the talking rate, defined as the proportion of time spent on talking during close contact. Based on previous studies, the talking rate of patients in general hospital wards was 23.2 % (Zhang, Liu, Dou, et al., 2023), which was assumed for P1. As P2 underwent endotracheal intubation, the talking rate was set to 0. The talking rate for P3 was calculated as 65.4 % based on video analysis. Additionally, because of the intubation procedure during the treatment of P2, the viral generation rate ($g_{b}$ and $g_{t}$) for P2 was amplified 60 times to account for the higher viral load in critically ill patients (Liu, Yan, et al., 2020).

#### 2.3.2 Long-range airborne transmission

For long-range airborne transmission, we assumed that the virus was uniformly mixed in the air. The total viral load in the room at time t, Q(t) (viral RNA load), could be calculated as follows:

(3) $$Q\left(t\right)=\sum_{s}\left[g_{b,s}\left(1-\theta \right)+g_{t,s}\cdot \theta \right]-V_{E}-\left(\frac{ACH}{3600}+\frac{p\cdot n}{3600\cdot V}+\gamma \right)\cdot Q\left(t-1\right)$$

where ACH represents the air changes per hour (h^−1^), the standard fresh air volume was set at 40 m^3^ per person per hour(Code for Design of General Hospitals*. (GB51039-2014)*). Given the room volume of 364 m^3^ and the presence of three patients and five HCWs, the air changes rate was about 1 ACH.) Most international guidelines recommend setting the air changes rate to 6 ACH for high-risk areas such as emergency rooms and intensive care units (Saran et al., 2020). *p* is the pulmonary ventilation rate, set at 0.38 m^3^/h (Chen et al., 2006); *n* is the number of individuals in the room; *V* is the room volume (364 m^3^); and *γ* is the virus inactivation rate, set at 0.66 h^−1^ (Van Doremalen et al., 2020).

The exposure rate via long-range airborne route, $E_{L}\left(t\right)$ (viral RNA load/s), is:

(4) $$E_{L}\left(t\right)=\frac{p\cdot Q\left(t\right)}{3600\cdot V}$$

#### 2.3.3 Effect of masks on exposure

Since patients receiving treatment cannot wear personal protective equipment (PPE), this study only focused on healthcare workers when analyzing the effectiveness of interventions. This study primarily considered the effects of N95 respirators and surgical masks on the spread of the epidemic. The inward protection efficiencies of N95 masks and surgical masks for fine aerosol were 94.1 % and 51.9 %, respectively (Koh et al., 2022). For large droplets, we assumed that both masks provided 100 % filtration efficiency (Liu et al., 2022). Therefore, the close contact exposure rate $E_{M}$ under different mask-wearing rates can be calculated as:

(5) $$E_{M}=V_{E}\cdot \left(1-\rho_{out}\right)\left(1-\beta \cdot \rho_{in}\right)$$

where $\rho_{in}$ and $\rho_{out}$ represent the inward and outward protection efficiencies, respectively, and β denotes the mask wearing rate, which was set according to the real value based on video analysis.

### 2.4 Infection risk assessment

Due to the lack of dose-response parameters relevant to SARS-CoV-2, and considering the similarity between SARS-CoV-2 and SARS-CoV (Mizukoshi et al., 2021), this study utilized the dose-response parameters of SARS-CoV.

Watanabe et al. (Watanabe et al., 2010), experimentally found that the exponential model p=1−exp(−d/k) can effectively predict the dose-response relationship for SARS-CoV. In this model, p represents the risk of infection, d represents the viral exposure, and k is a pathogen-specific parameter. Therefore, the probability of infection p due to the accumulated viral exposure over a period of time for a susceptible individual can be calculated using the following equation:

(6) $$p=1-\exp\left(-\frac{D_{SI}+D_{LI}}{k_{i}}-\frac{D_{DP}}{k_{d}}\right)$$

where $D_{SI}$, $D_{DP}$ and $D_{LI}$ represent the viral exposure (viral RNA load) through short-range inhalation, facial mucous deposition, and long-range airborne transmission, respectively. $k_{i}$ and $k_{d}$ represent the dose-response parameters for respiratory and facial mucous, respectively. Based on previous studies, $k_{i}$ and $k_{d}$ were set to 40.65 viral RNA load and 406.5 viral RNA load, respectively (Zhang, Zhao, et al., 2024).

Relative contribution rate, which is defined as the ratio of the infection risk from a specific transmission route to the total infection risk, was also calculated using the dose-response model.

When processing data, variables that follow a normal distribution were described using the mean ± standard deviation. For variables that do not follow a normal distribution, the median (25th percentile, 75th percentile) was reported.

## 3 Results

This study analyzes a total of 19,246 s of video data captured in an emergency room setting. The average durations of a single treatment episode were 1409 s for P1 (infusion patient), 1035 s for P2 (critically ill patient), and 764 s for P3 (agitated patient).

### 3.1 Close contact behaviors

**Fig. 5:**
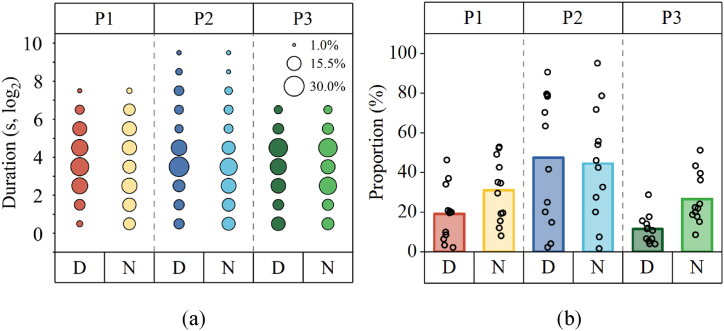
Close contact behaviors between HCWs and patients. (a) Distribution of duration per close contact; (b) close contact rate (bars represent the mean values, while the circles represent the values of each HCW). D and N denote doctor and nurse, respectively.

The durations of close contact for HCWs when treating patients P1, P2, and P3 are 383.5 (327, 752) seconds, 874.5 (716, 1060) seconds, and 181.5 (58.6, 453.5) seconds, respectively (Fig. 5a). There was no significant difference in the close contact duration between “doctors and patients” and “nurses and patients” (p = 0.88).

When treating P2, the close contact rate is the highest (46.0 %), for both doctors (47.5 %) and nurses (44.5 %), significantly exceeding the rate observed during the treatment of P1 (25.0 %, p < 0.001) and P3 (19.0 %, p < 0.001) (Fig. 5b). During the treatment of P1 and P3, the close contact rate for nurses was 1.6 times and 2.3 times higher than that for doctors, respectively.

**Fig. 6:**
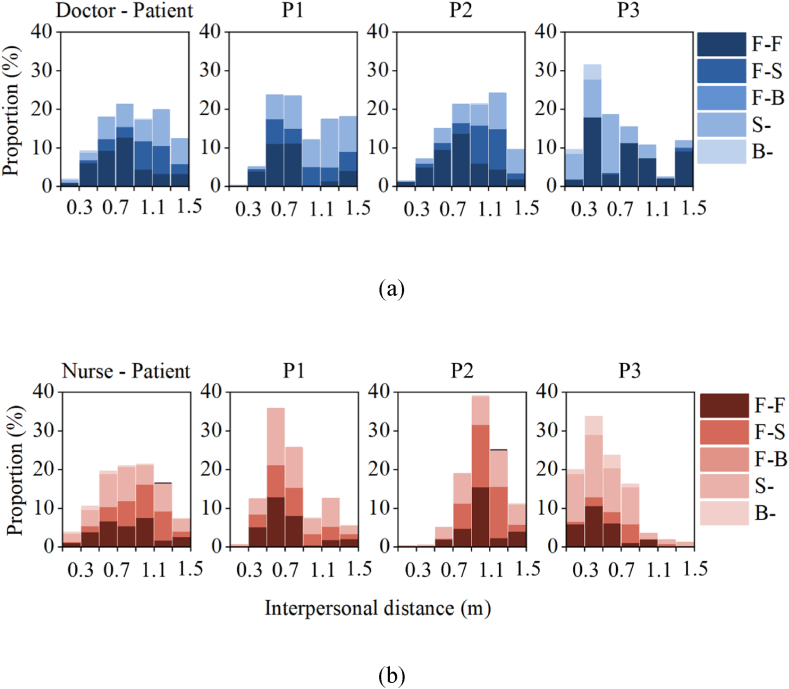
Probability distribution of interpersonal distance and relative facial orientation. (a) Doctor-Patient; (b) Nurse-Patient. (F–F: face-to-face; F–S: face-to-side; F–B: face-to-back; S-: indicates side of the infected; B-: indicates back of the infected.)

The average interpersonal distance between nurses and patients (0.89 m) was slightly shorter than that between doctors and patients (0.95 m) (Fig. 6). During close contact, doctors were most likely to engage in face-to-face (F-F) contact with patients (40.4 %), while contact with the side of the infected (S-) and the back of the infected (B-) was rare (less than 1 %). Nurses were more likely to engage in the side of the infected (S-) contact with patients (39.3 %), with face-to-back (F-B) contact being almost nonexistent (0.1 %). Across all patient treatments (P1, P2, and P3), the proportion of face-to-face contact between doctors and patients exceeds that between nurses and patients, being 1.1, 1.4, and 2.0 times higher, respectively. This may be attributed to the fact that doctors need to observe patients closely and record their conditions for extended periods.

For three patients, the shortest average interpersonal distance was observed during the treatment of P3 (0.5 m), with the average distances of 0.73 m and 0.57 m for doctors and nurses, respectively. In contrast, during the treatment of P1 and P2, the average distance between nurses and patients is 0.82 times and 1.10 times that between doctors and patients, respectively. This difference may be due to the more frequent need for nurses to perform treatment operations on patients during the care of P1 and P3, resulting in closer contact. Conversely, during the treatment of P2, doctors took the lead in performing the critical procedures, resulting in a shorter interpersonal distance of 0.97 m between doctors and patients compared to 1.07 m between nurses and patients.

### 3.2 Viral exposure and infection risk

#### 3.2.1 Viral exposure

**Fig. 7:**
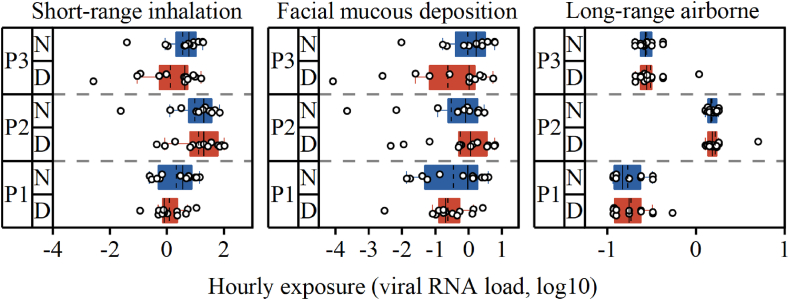
Hourly exposure. (Circles represent the value for HCWs; Solid line indicates the median, dashed line indicates the mean, ends of the box represent the 25 and 75 percentiles.)

The virus generation rate during the treatment of P2 was 60 times higher than that of other patients. Consequently, the viral exposure experienced by HCWs during the treatment of P2 was 7.0 and 4.0 times higher than it during the treatment of P1 and P3, respectively. Furthermore, the viral exposure experienced by doctors was significantly higher than that of nurses, with doctors experiencing 1.4 and 1.9 times higher short-range inhalation and facial mucous deposition exposure, respectively, compared to nurses (Fig. 7). In contrast, during the treatment of P1 and P3, nurses experienced 2.0 and 1.6 times higher short-range inhalation and 2.1 and 1.7 times higher mucous deposition exposure, respectively, compared to doctors.

#### 3.2.2 Infection risk

**Fig. 8:**
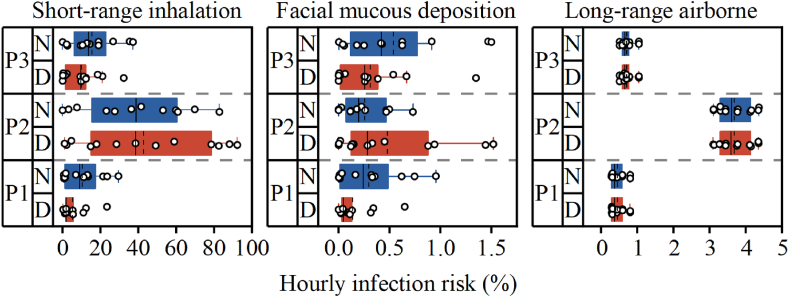
Hourly infection risk (%). (Circles represent the value for HCWs; Solid line indicates the median, dashed line indicates the mean, ends of the box represent the 25 and 75 percentiles.)

In the absence of mask wearing, the average hourly infection risks for HCWs in the emergency room during the treatment of P1, P2, and P3 were 8.5 %, 43.4 %, and 13.9 %, respectively (Fig. 8). The primary transmission route was short-range airborne transmission, contributing 78.2 % to the total infection risk, followed by long-range airborne transmission at 20.3 %, while large droplets transmission contributed less than 2 %. During the treatment of P1, nurses faced an infection risk 1.8 times higher than that of doctors, likely due to their primary role in performing infusion procedures. No significant difference in infection risk was observed between doctors and nurses during the treatment of P2 (p > 0.05). However, during the treatment of P3, nurses faced an infection risk 1.5 times higher than that of doctors, possibly because nurses took the lead in assisting the agitated patient, resulting in closer contact.

**Fig. 9:**
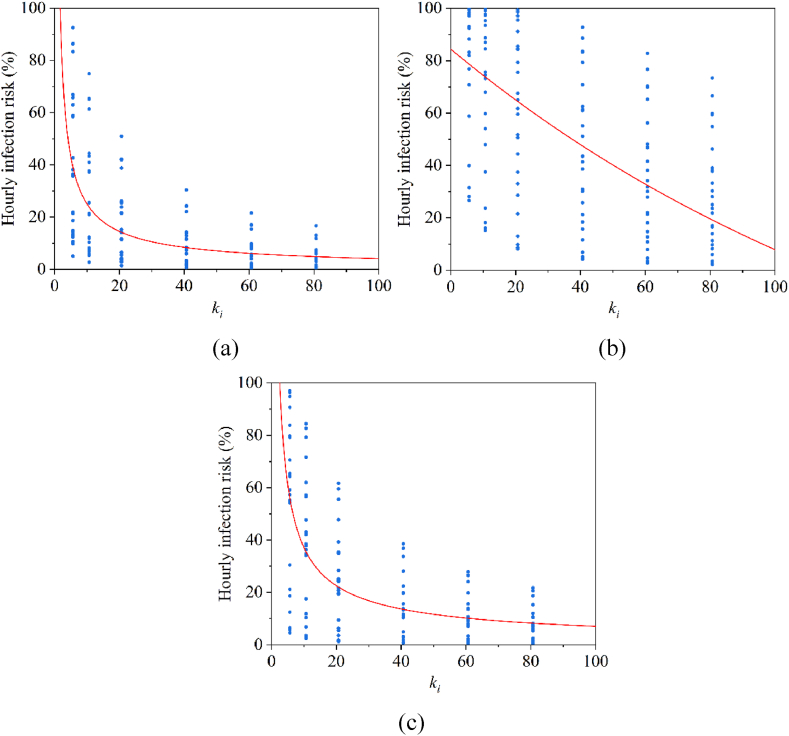
The relationship between the parameter of dose-response model and the hourly infection risk ($k_{i}/k_{d}=0.1$) when treating (a) P1, (b) P2, and (c) P3 (blue dots are values for all HCWs).

This study has conducted a sensitivity analysis of the $k_{i}$ value (Fig. 9). The results indicated a negative correlation between the total infection risk and the k value, with the risk decreasing progressively as the $k_{i}$ value increased. It was noteworthy that the trends in infection risk vary significantly across different treatment scenarios. Specifically, when treating P1 and P3 patients, the infection risk trends were largely similar, however, in the case of P2 patients, the infection risk curve exhibited a slower rate of change. This difference may be attributed to the substantially higher baseline infection risk faced by healthcare workers when treating P2 patients, as compared to those treating P1 or P3 patients, thereby limiting the effect of changes in the $k_{i}$ value on the overall risk.

### 3.3 Non-pharmaceutical interventions

#### 3.3.1 Mask

**Fig. 10:**
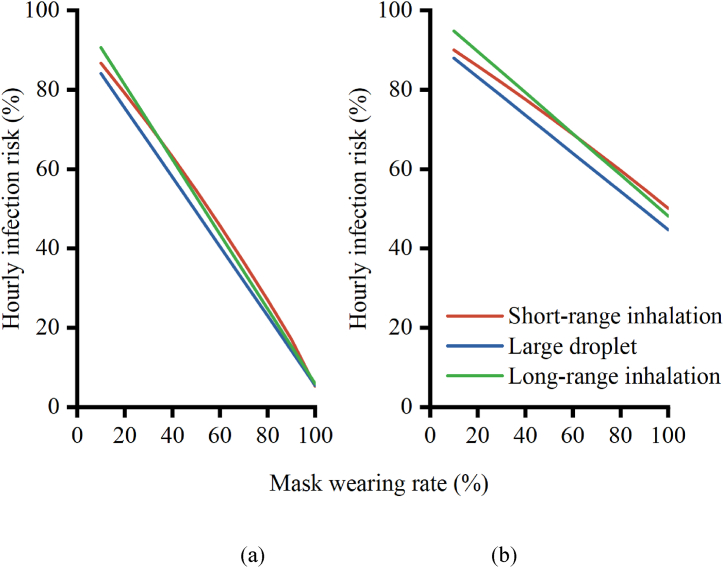
The relationship between mask wearing rate and infection risk: (a) N95 respirator; (b) Surgical mask.

If all HCWs wore N95 masks during treatment, the total hourly infection risk would decrease by 94.7 % (Fig. 10). Specifically, the infection risks associated with short-range airborne, large droplets, and long-range airborne transmission would be reduced by 94.7 %, 94.5 %, and 94.1 %, respectively. If all HCWs wore surgical masks, the total hourly infection risk would be reduced by 53.9 %.

#### 3.3.2 Indoor ventilation

**Fig. 11:**
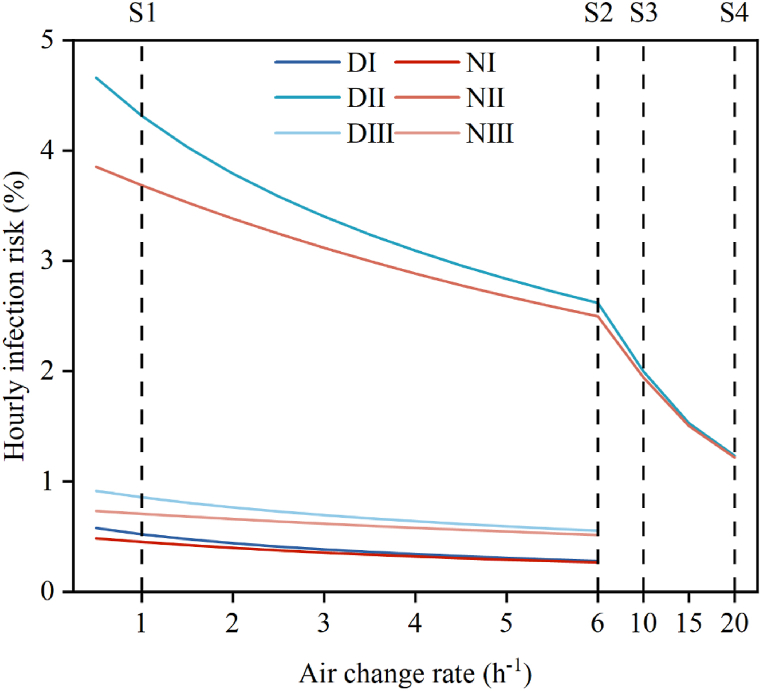
The variation of infection risk from long-range airborne transmission with indoor air change rates, D(N)Ⅰ-Ⅲ represents the average value of all doctors (nurses) treating P1-3. S1 represents the indoor ventilation standard for emergency rooms in China (Code for Design of General Hospitals*. (GB51039-2014)*); S2 is the indoor ventilation standard for emergency rooms in the US (Ventilation of Health Care Facilities); S3 represents the ventilation standard for operating rooms in the UK (Saran et al., 2020); S4 represents the ventilation standard for operating rooms in the US (Saran et al., 2020).

When the ventilation rate was set to the national standard of 40 m^3^/person/h, the air changes rate was approximately 1 ACH. Increasing the air changes rate from 1 ACH to 6 ACH reduced the infection risk associated with long-range airborne transmission by 43.8 % for P1, 36.1 % for P2, and 31.6 % for P3 (Fig. 11). On average, the infection risk for doctors and nurses decreased by 40.3 % and 33.4 %, respectively. When the air change rate was further increased to the standard recommended for operating rooms (10–20 ACH), the infection risk could be reduced by 50.5 %–69.2 %.

## 4 Discussion

This study collected and analyzed video data from an emergency room within a medical school in Hong Kong, focusing on the close contact behaviors between HCWs and patients. Using COVID-19 as an example, we developed a multi-route transmission model considering real close contact behaviors between HCWs and patients. Then, the viral exposure, infection risk, and the effectiveness of mask wearing and indoor ventilation were assessed.

Human behavior plays a critical role in the transmission of COVID-19 (Azuma et al., 2020). We found that the average close contact rate between HCWs and three patients in the emergency room reached 30.0 %, and it was the highest (46.0 %) during the treatment of P2 (critically ill patient), which is significantly higher than in other indoor environments. For example, the close contact rate was 10.0 % in an office setting (Zhang et al., 2020), 44.5 % in university classroom (Zhang, Yang, et al., 2024) and 12.7 % in homes (Zhang, Liu, Dou, et al., 2023). The high close contact rate in emergency rooms, coupled with a high disease incidence, presents unique challenges for infectious disease control. Because treating critically ill patients requires continuous medical intervention and constant monitoring of vital signs, the close contact rate was the highest during P2 treatment. During the treatment of P1 (infusion patient) and P3 (agitated patient), nurses exhibited a significantly higher close contact rate compared to doctors, indicating that personal protection equipment of nurses should be give more attention.

Using COVID-19 as an example, the viral generation rate during talking is nearly 20 times higher than it during breathing (Coleman et al., 2021). When the talking rate is 10 %, the exposure risk doubles; when it is 20 %, the exposure risk quadruples (Park & Kim, 2021). In this study, the talking rates for P1 and P3 were 23.2 % and 65.4 %, respectively. Due to the high viral load of SARS-CoV-2 found in the sputum and upper respiratory secretions of COVID-19 patients, tracheal intubation should be considered as a high-risk procedure for the transmission of SARS-CoV-2 (Weissman et al., 2020). During the COVID-19 epidemic, a large number of patients require invasive ventilation and intubation (van der Ven et al., 2024), so the risk of infection faced by HCWs from such patients (P2) was greatly increased.

Interpersonal distance is a crucial factor in the transmission of respiratory infectious diseases (Cortellessa et al., 2021). The infection risk at a 2-m distance is about one-thousandth of that at 0.4 m during face-to-face close contact (Chen et al., 2020). We found that the average interpersonal distances between HCWs and the three patients were 0.8 m (P1), 1.0 m (P2), and 0.5 m (P3), respectively. Because nurses need to maintain closer contact with patients to perform treatment operation, during non-critical treatment (P1 and P3), the average distance between nurses and patients was significantly shorter than that between doctors and patients. However, during critical treatment (P2), doctors played a more important role, resulting in a shorter interpersonal distance between doctors and patients (0.97 m) compared to that between nurses and patients (1.07 m). The interpersonal distances observed in the emergency room were shorter than the average social distance of 1.2 m in China (Sorokowska et al., 2017), and is also shorter than the interpersonal distance observed in university classrooms (0.9 m), homes (0.95 m), postgraduate student offices (0.95 m), and shopping centers (0.92 m) (Zhang, Liu, Dou, et al., 2023). These shorter distances likely facilitate viral transmission and make infection control more challenging in the emergency room.

The relative facial orientation between HCWs and patients also impacts the risk of viral exposure. Aerosol exposures during talking and breathing are 12.0 % and 33.8 % in face-to-back contact compared to face-to-face contact, respectively (Wei et al., 2023). This study found that during the treatment of all three patients, doctors had a higher probability of face-to-face close contact with patients than nurses. The probability of face-to-face close contact was the highest during the treatment of P3, with doctors having a significantly higher probability (52.8 %) than nurses (26.0 %).

Due to the nature of the emergency room, controlling interpersonal distance and relative facial orientation between HCWs and patients are challenging, making personal protective measures essential. Wearing masks significantly reduces infection risk for susceptible individuals (Liu & Deng, 2023), if the mask-wearing rate remains at a high level, it can effectively reduce the transmission of infectious diseases (Nagata et al., 2024).The study found that if all HCWs wore N95 respirators and surgical masks during treatment, the total infection risk could be reduced by 94.7 % and 53.9 %, respectively. However, air leakage around the mask reduces its effectiveness (Solano et al., 2022), so ensuring a proper fit is crucial. The study also revealed that nurses faced a higher infection risk than doctors when treating infusion patients and agitated patients, emphasizing the need for stricter protective measures for nurses. Besides masks, HCWs should use appropriate PPE, such as goggles and face shields, and follow COVID-19 infection prevention and control (IPC) measures (Azimi et al., 2021) to further minimize the infection risk.

The study highlights that short-range airborne transmission is the dominant mode (78.2 %) in emergency rooms, followed by long-range airborne route (20.3 %). These findings align with those from ordinary wards, where close contact transmission accounts for 60 %–86 % of exposure risk under similar conditions (Mizukoshi et al., 2021). However, in other indoor environments, such as high-speed trains and university classrooms, long-range airborne transmission is the main route of spread (Zhang et al., 2023b, 2024b). This is due to the small indoor space, high occupant density, low face-to-face orientation, and uncertainty about who is infected, meaning fewer people are in close proximity to an infected person. This study reflects real emergency room scenarios, assuming that the patient is the source of infection and that all HCWs are in continuous close contact with the patient during treatment, making close contact transmission dominant.

In this study, increasing indoor ventilation had limited effect in reducing the risk of infection. The standard air change rate in the emergency room was about 1 ACH *GB51039-2014*). When the air change rate increased to 6 ACH, the infection risk of long-range airborne transmission decreased by 36.9 %. Considering main contribution of short-range airborne route, interventions such as wearing masks could effectively mitigate close contact transmission, while increasing ventilation has less impact on reducing overall infection risk.

This study has some limitations. Firstly, we only assessed the infection risk for HCWs when the patient was the source of infection, without considering the scenario where HCWs are the index patient. Secondly, P1 (infusion patient) and P2 (critically ill patient) were mannequins, which influenced the patterns of close contact behaviors. Additionally, the video used was from a clinical skills competition at a medical school, not from an actual emergency room, which reduces the reliability of the results. However, due to the privacy, the surveillance video in real emergency rooms was almost impossible to obtained. Thirdly, the data volume in this study was not large, which may lead to limited reliability. Fourth, when assessing long-range airborne transmission, it is assumed complete air mixing, which could introduce some errors.

## 5 Conclusions

This study provides valuable insights into the transmission dynamics of respiratory infectious diseases in emergency rooms based on real close contact behaviors between HCWs and patients. In this study, short-range airborne transmission was the primary mode of transmission in the emergency room (78.2 %), followed by long-range airborne transmission (20.3 %). Close contact between HCWs and patients significantly affected the risk of respiratory infectious diseases, especially when treating critically ill patients (e.g., during intubation), where the infection risk reached 43.4 %/h without masks. For non-critical patients (infusion and agitated patients), nurses experienced both longer close contact times and higher infection risks compared to doctors. Wearing N95 masks reduced the overall infection risk for HCWs by 94.7 %, while surgical masks decreased the risk by 53.9 %. Increasing the ventilation rate from 1 ACH to 6 ACH can lower the overall infection risk by approximately 3 %, with a more significant effect of up to 5.6 % when treating intubated patients.

## CRediT authorship contribution statement

**Bing Cao:** Writing – review & editing, Writing – original draft, Visualization, Methodology, Investigation, Formal analysis, Data curation. **Haochen Zhang:** Writing – original draft, Visualization, Validation, Formal analysis, Data curation. **Nan Zhang:** Writing – review & editing, Writing – original draft, Visualization, Methodology, Investigation, Funding acquisition, Formal analysis, Data curation.

## Ethics approval and consent to participate

This study was approved by the Ethics Committee for Science and Technology, Beijing University of Technology (Approval number: CJXB12).

## Consent for publication

All participants in this experiment consented to publication.

## Declaration of competing interest

The authors declare that they have no known competing financial interests or personal relationships that could have appeared to influence the work reported in this paper.
